# Psychological Burden During the COVID-19 Pandemic in Germany

**DOI:** 10.3389/fpsyg.2021.640518

**Published:** 2021-09-07

**Authors:** Iris Schelhorn, Angelika Ecker, Michael Noah Lüdtke, Stefan Rehm, Thomy Tran, Judith Lena Bereznai, Marie Lisa Meyer, Stefan Sütterlin, Max Kinateder, Ricardo Gregorio Lugo, Youssef Shiban

**Affiliations:** ^1^Department of Educational Psychology, University of Regensburg, Regensburg, Germany; ^2^Clinic and Polyclinic for Child and Adolescent Psychiatry, Psychosomatics and Psychotherapy, University of Regensburg, Regensburg, Germany; ^3^Department of Psychology, Private University of Applied Sciences, Göttingen, Germany; ^4^Faculty of Health and Welfare Sciences, Østfold University College, Fredrikstad, Norway; ^5^National Research Council Canada (NRC-CNRC), Ottawa, ON, Canada; ^6^Faculty of Social and Health Sciences, Inland Norway University of Applied Sciences, Elverum, Norway

**Keywords:** COVID-19, psychological burden, Germany, depression, mental health

## Abstract

After the first COVID-19 case was diagnosed in Germany, various measures limiting contact between people were introduced across the country. The implementation of these measures varied between jurisdictions and potentially had a negative impact on the psychological well-being of many people. However, the prevalence, severity, and type of symptoms of psychological burden has not been documented in detail. In the current study, we analysed various self-reported symptoms of psychological burden in a German sample. The dataset was collected between April 8th and June 1st, 2020, through an online survey measuring psychological burden using the ICD-10-symptom rating scale. More than 2,000 individuals responded to the survey, with a total of 1,459 complete datasets. Data was then sampled to compare (1) the new data to an existing demographically comparable reference dataset including a total of 2,512 participants who did not undergo any kind of contact restrictions or other pandemic measurements, and (2) psychological burden in two different German states. In line with recent observations from Germany, Italy, China, Austria and Turkey, we found a high prevalence of depressive symptoms in comparison to the reference sample. Furthermore, we found a high prevalence of eating disorder and compulsion symptoms. Especially younger adults and women reported a higher symptom severity compared to other groups during our measurement period. However, no difference between the two states in psychological burden was found.

## Introduction

The World Health Organization (WHO) declared a public health emergency of international concern on 30 January 2020 due to the COrona VIrus Disease 2019 (COVID-19). Such a declaration implies that a disease can potentially have a serious impact on public health, including mental health [World Health Organization (WHO), 2020]. Because of this declaration, many governments enacted public health interventions such as physical distancing, canceling leisure time activities, mandatory breaks for schools and universities, travel restrictions and obligatory quarantine for anyone tested positive for the disease. Some of these measures restricted personal movement and may therefore have led to social isolation. Both have been linked to an increase in stress-responses and even mortality rates (Grewen et al., 2003; Cacioppo et al., 2015; Holt-Lunstad et al., 2015; Conradi et al., 2020). The meta-analytic study of Holt-Lunstad et al. (2015) found increases in mortality rates of 32% for living alone, 26% for loneliness and 29% for social isolation. In addition, the economic consequences of the restrictions have begun taking their toll, for instance, through a sharp increase in unemployment and decreased employment security (e.g., in Germany). The economic consequences are likely to further amplify the psychosocial burden, given that loss of employment, for instance, is a highly stressful life event (Slavich and Shields, 2018). Therefore, it is not surprising that the prevalence of depression, but also of other mental disorders, has increased since the beginning of the COVID-19 pandemic and the implementation of measures to restrict social contact (e.g., Tang et al., 2020).

Recent studies concerning the psychological effects of the COVID-19 pandemic worldwide have shown an increased risk in mental disorders in different countries. Pieh et al. (2020) observed a prevalence of 21% for depression symptoms and 19% for anxiety symptoms in Austria over a 2-week period (until April 30th, 2020) following a 4-week lockdown due to the Covid-19 pandemic. Comparable results were demonstrated in Italy (between March 27th and April 06th 2020) with the prevalence of disorder symptoms being 17.3% for depression and 20.8% for anxiety symptoms (Rossi et al., 2020). Wang et al. (2021) reported a prevalence of 17% for depression as well as a prevalence of 6% for anxiety symptomatology (from February 6 to February 9 2020) in a Chinese sample. In Turkey (between April 14 and April 16 2020) results show that 23% of the participants scored above the depression cut-off and 45% scored above the anxiety cut-off of the Hospital Anxiety and Depression Scale (Özdin and Bayrak Özdin, 2020). Similar results were found in Switzerland where de Quervain et al. (2020) investigated the impact of the COVID-19 pandemic on mental health where over half of the participants reported an increase in depressive symptoms during confinement compared to before the virus outbreak. In this study, those with a history of psychiatric disorders were more at risk than participants with no prior psychiatric issues whereas older people and men were more resilient. Other studies confirm these findings and also show that female participants had higher depression scores than men, were lonelier and suffered more from daily life fatigue (Bartoszek et al., 2020). Specifically for the German population, studies indicate comparable effects of the COVID-19 situation and the accompanying restrictions on mental health. A large study showed high prevalence for depressive symptoms, generalized anxiety disorder and general distress (Bäuerle et al., 2020b) and several studies indicate that the psychological burden is especially high for women (Bäuerle et al., 2020a; Peters et al., 2020; Petzold et al., 2020). Furthermore, the pandemic and the restrictions it entailed seemed to represent a greater psychological burden for younger compared to older people (Bäuerle et al., 2020b; Peters et al., 2020). Specifically, a study of Röhr et al. (2020) showed older people (> 65 years) to be more resilient regarding depression, anxiety and somatization symptoms. Another recent study focusing on health care staff in Germany showed that health care workers were less psychologically burdened than the comparison group regarding symptoms of depression, anxiety and fear of COVID in the first weeks of restrictions (Skoda et al., 2020).

In the present study, psychological well-being during the time of restrictions following the COVID-19 outbreak was explored in different regions of Germany, mainly Bavaria and Lower Saxony. This could be of interest, as due to the federal state structure of Germany and differences in impact of COVID-19, German states imposed different levels of curfew, while other factors influencing stress and well-being under a pandemic such as healthcare system infrastructure, availability and accessibility of healthcare systems (Brooks et al., 2020), are comparable across Germany. Furthermore, sociodemographic structures, economic situations, and trust in public authorities are equally comparable in all states. We explored psychological burden shortly after restrictions were introduced and compared them to a reference sample from 2010 (Tritt et al., 2010) containing non-clinical participants who did not undergo any kind of contact restrictions or other pandemic related measures. We were interested in different mental health syndromes: depression, anxiety, somatization, eating disorder symptoms, and compulsion. We hypothesized that there would be a high prevalence of depressive symptoms due to an increase in stress level. Furthermore, we hypothesized that the measures resulted in different levels of psychological stress and therefore reported symptoms in two different regions expecting higher levels in Bavaria than in Lower Saxony as the restrictions were more intense in the Bavarian region.

## Materials and Methods

### Study Design

**FIGURE 1:**
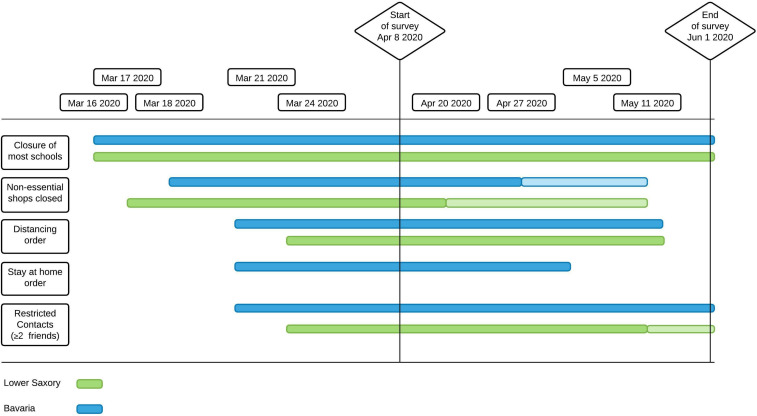
Timeline for restrictions in Lower Saxony and Bavaria. *Closure of most schools* = Some classes with special priorities could still attend school (e.g., graduating classes); *Stay at home order* = People are not allowed to leave the house except for work, shopping essentials or walking/jogging alone. At the beginning of June 2020, in Bavaria social contacts were still restricted by order (less than 2 contacts allowed), whereas in Lower Saxony there was only a recommendation.

A cross-sectional design was used to collect survey data in a web-based questionnaire. The effects of age, gender, and region of residence on severity of symptoms were further explored. The 15-min survey included additional questionnaires; however, those characteristics were outside the scope of the present analysis. The data was collected between April 8 and June 1 2020. The timeline of this study within the context of COVID-19 development is depicted in Figure 1.

### Sample

Participants were recruited via flyers, social online platforms, mailing lists and notices in in-patient clinics and supermarkets. Out of 2,506 participants who had started the questionnaire, 1,739 datasets (69.4% valid sets; 72.6% female) contained data that was usable for statistical analysis for this manuscript. We excluded 280 participants who reported to have been or were in psychotherapy, as the sample we used for comparison (reference sample) was a mentally healthy group of individuals. The resulting sample size was *N* = 1,459 (71.4% females), the age is ranged between 18 and 88 years, *M* = 34.35, *SD* = 14.04. Exclusion criteria were an age younger than 18 years, unrealistic or missing values as well as not having completed the sections needed for this analysis. Demographic data are depicted in Table 1. An indication of the e-mail address for future investigations was voluntary, otherwise no further personally identifiable information was collected. All participants gave their informed consent for participation and completed the questionnaires electronically. Data was collected anonymously without IP addresses or GPS tracking. Email address, when provided, was separated from the rest of the dataset. The study was approved by the Ethics Committee of the Department of Psychology at the PFH – Private University of Applied Sciences (Ethics application number: 251982).

**TABLE 1:** Demographics of total survey sample and resampled survey sample.

Variable	Total		Resampled	
	survey sample		survey sample	
	*n*	%	*n*	%
Total	1459	100	875	100
**Gender**				
Male	417	28.6	417	47.7
Female	1042	71.4	458	52.3
**Marital Status**				
Single	886	60.7	555	63.4
Married	477	32.7	264	30.2
Widowed	22	1.5	13	1.5
Separated	74	5.1	43	4.9
**Living Situation**				
Alone	205	14.0	124	14.2
Shared Flat	176	12.1	108	12.3
With partner	426	29.2	256	29.3
With family	652	44.7	387	44.2
**Job Status during lockdown**				
Attending work as usual	390	26.7	246	28.1
Attending work in home-office	481	33.0	293	33.5
Mixture	323	22.1	190	21.7
Not working	143	9.8	86	9.8
Question does not apply to situation	122	8.4	60	6.9
**Living Area**				
Urban	604	41.4	380	43.4
Rural	855	58.6	495	56.6
COVID-19 diagnosis	10	0.7	6	0.7
COVID-19 diagnosis in friends/family	439	30.1	259	29.6
Quarantined	85	5.8	48	5.5

#### Reference Sample

For standardization of the ISR questionnaire, which is described in the measures section, Tritt et al. (2010) used a sample of *n* = 8,892 in-patients and of *n* = 2,512 non-clinical subsample from all over Germany. Therefore, the non-clinical subsample is comparable to our sample as it has also been recruited throughout Germany. For this reason, we used it in the subsequent analysis and will refer to it as the “reference sample “. In contrast to our sample, the participants of Tritt et al. (2010) did not undergo any kind of contact restrictions or other pandemic measurements.

#### Data Reduction: Subsamples

**FIGURE 2:**
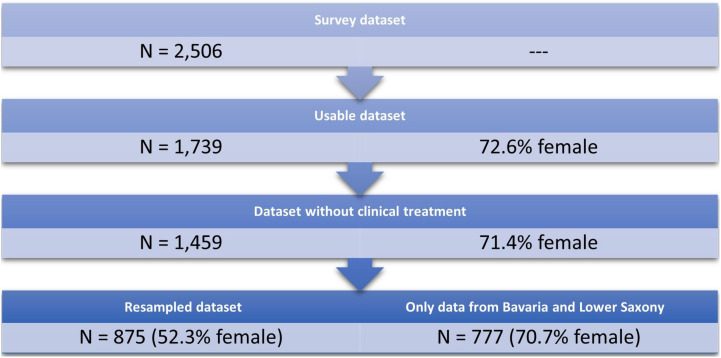
Resampling procedure. From the remaining 1459 participants, 875 were resampled in order to balance the gender distribution.

To answer distinct research questions, two subsamples were used: The full sample consisted of 417 male and 1,042 female participants. We first matched our data to the existing reference sample (Tritt et al., 2010) in order to understand the magnitude of psychological burden in our data as a whole. In order to compare our data to a reference sample, 458 female participants were randomly selected to adapt the gender distribution of our sample to the reference sample, resulting in a subsample of *n* = 875 participants (52.3% female). For comparing states with different lockdown measures, we only analysed data from participants living in Bavaria and Lower Saxony and excluded participants who lived outside the two target states, yielding a total of 777 complete data sets. The sampling procedure is depicted in Figure 2.

### Measures

#### Depressive Symptoms and Mental Health

Depression and other mental health symptoms were both measured with the respective subscale of the self-report questionnaire ICD-10-Symptom-Rating [ISR, Tritt et al. (2008)] that can be used for clinical diagnostics in the German-speaking area. The ISR was designed and validated for the rapid assessment of depression, anxiety, eating disorder, obsessive compulsive disorder, and somatoform disorder symptoms. In total, the ISR contains 29 items to be rated on a 5-point-Likert-scale from 0 to 4, with 0 indicating “does not apply” and 4 indicating “extremely applies.” The period under consideration is the last 2 weeks. Subscales consist of either three (eating disorder subscale: e.g., “I spend a lot of time thinking of ways to lose weight”) or four items (e.g., depression subscale: e.g., “I feel down and depressed”). There are also supplementary items (12 items) focusing on other ICD classifications, that are not included in the above categories. Furthermore, we added three items measuring reported differences in substance consumption. The ISR item scores are averaged for each subscale and the subscales can be taken together in a total score. The average of the response values of a category provides the score for each individual subscale, which is categorized according to severity. For instance, symptomatology of depression was considered suspicious, mild, moderate or severe if the respective average score was ≥0.75, ≥1.0, ≥2.0 or ≥3.0. The reported internal consistency for the total score is very good (Cronbach’s α = 0.92) with slightly lower scores for the subscales (Cronbach’s α = 0.78 – 0.86). The strengths of the ISR is its brevity and its pragmatic approach to good quality criteria, with validated scales: The ISR has shown good associations to other validated diagnostic tools such as the PHQ-D [German version of “PRIME MD Brief Patient Health Questionnaire,” Löwe et al. (2002)] and with the widely used symptom Checklist-90 R [SCL-90; Franke and Derogatis (2002)] and has been validated in clinical samples (e.g., Brandt et al., 2015). In our sample, we calculated Cronbach’s α for the depression subscale (α = 0.81), the anxiety subscale (α = 0.85), the compulsion subscale (α = 0.83), the somatization subscale (α = 0.83) and the eating disorder subscale (α = 0.81).

#### Lockdown Severity

To compare different degrees of severity of the measures taken, we compared the data gained from the federal German states of Lower Saxony and Bavaria, as both states had enforced lockdown measures differing in severity (see Figure 1), based on the lockdown measures dataset from Steinmetz et al. (2020). In Bavaria, obligatory restrictive curfew was imposed starting from 21th of March until the beginning of May: People were only allowed to leave their houses to take care of the absolutely necessary (e.g., in certain cases going to work, doing sports on their own, grocery shopping and attending to home care services). In Lower Saxony, starting from 23rd of March, social distancing was recommended and enforced, however, citizens were only advised to voluntarily stay at home if possible.

### Statistical Analysis

The subsequent analysis focused on comparing our sample and the reference sample. When comparing means between independent groups we calculated Welch’s *t*-tests, due to inhomogeneity of variance between samples. As a measure for effect size, we used Hedge’s *g* due to a large difference in sample size. Furthermore, for depressive symptoms, we explored differences between male and female participants, age groups and states. In a series of hierarchical regression models, combinations of age, gender and state were used to predict the average score of depression, compulsion, anxiety, eating disorder and somatization symptoms. To correct for multiple comparisons where appropriate, the false discovery rate [FDR, Benjamini and Hochberg (1995)] was applied. Reported *p*-values reflect the FDR correction. The statistical analysis was carried out using R 3.6.2 (R Core Team, 2017).

## Results

### Specific Symptoms

The items with the highest mean scores in the survey sample were “I feel down and depressed” (*M* = 1.25, *SD* = 1.04) for the depression subscale, “I try to avoid these harmless frightening situations” (*M* = 0.76, *SD* = 1.09) for the anxiety subscale, “I try to resist recurring, seemingly senseless thoughts and actions, but often don’t succeed” (*M* = 0.54, *SD* = 1.00) for the compulsion subscale, “I worry about having a serious physical illness” (*M* = 0.37, *SD* = 0.80) for the somatization subscale and “I spend a lot of time thinking of ways to lose weight” (*M* = 0.90, *SD* = 1.21) for the eating disorder subscale.

### Comparison of the COVID-19 Sample With the Reference Sample

**FIGURE 3:**
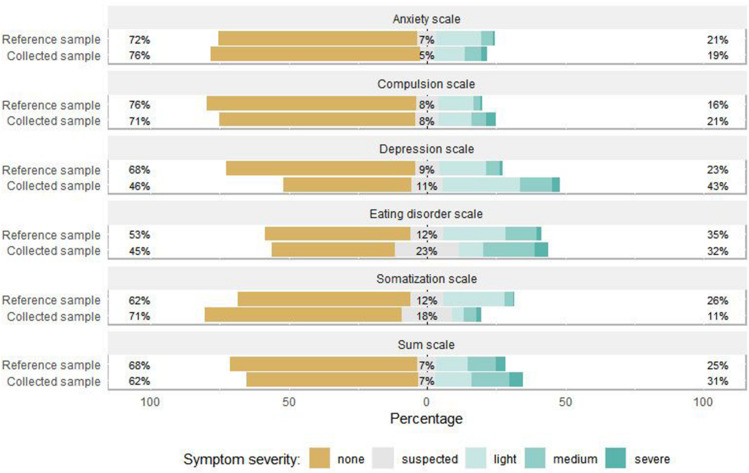
Symptom severity percentages for survey sample and reference sample. The percentage (%) on the right refers to the combined percentage of light, medium and severe symptom severity. This figure illustrates data from the survey sample (*N* = 875) and the reference sample (*N* = 2512).

We found significant differences between the reference and the COVID-19 sample in depressive symptoms, compulsive symptoms and symptoms of eating disorder, as displayed in Table 2 and Figure 3, with higher burden in the COVID-19 sample. We found no different scores on the anxiety scale and a very small difference in somatization symptoms with higher prevalence in the reference sample (see Table 2). Significant medium effects for depressive disorder and significant but small effects for compulsive, eating disorder and somatization symptoms were found.

**TABLE 2:** Mean and Standard Deviation of ISR subscale scores for reference sample and survey sample.

	Sample					Effect size
	Reference sample (*N* = 2512)	Survey sample (*N* = 875)				
					
ISR Scales	*M* (*SD*)	*M* (*SD*)	*df*	*t*	*p*	*g*
Depression	0.54 (0.69)	0.91 (0.83)	1318	11.84	0.006**	0.51
Anxiety	0.45 (0.66)	0.46 (0.77)	1348	0.34	0.732	0.01
Compulsion	0.32 (0.57)	0.47 (0.81)	1189	5.06	0.003**	0.23
Eating disorder	0.52 (0.76)	0.71 (0.95)	1285	5.35	0.002**	0.23
Somatization	0.35 (0.60)	0.27 (0.64)	1444	–3.24	0.001**	0.13
Sum	0.40 (0.45)	0.53 (0.55)	1304	6.30	0.001**	0.27

Means (*M*) and standard deviations (SDs) for the ISR subscales; *p* = adjusted significance (α < 0.05); ** indicates *p* < 0.01; effect sizes are reported as Hedge’s *g*.

### Gender, Age and State of Residence and ISR Symptom Severity

**FIGURE 4:**
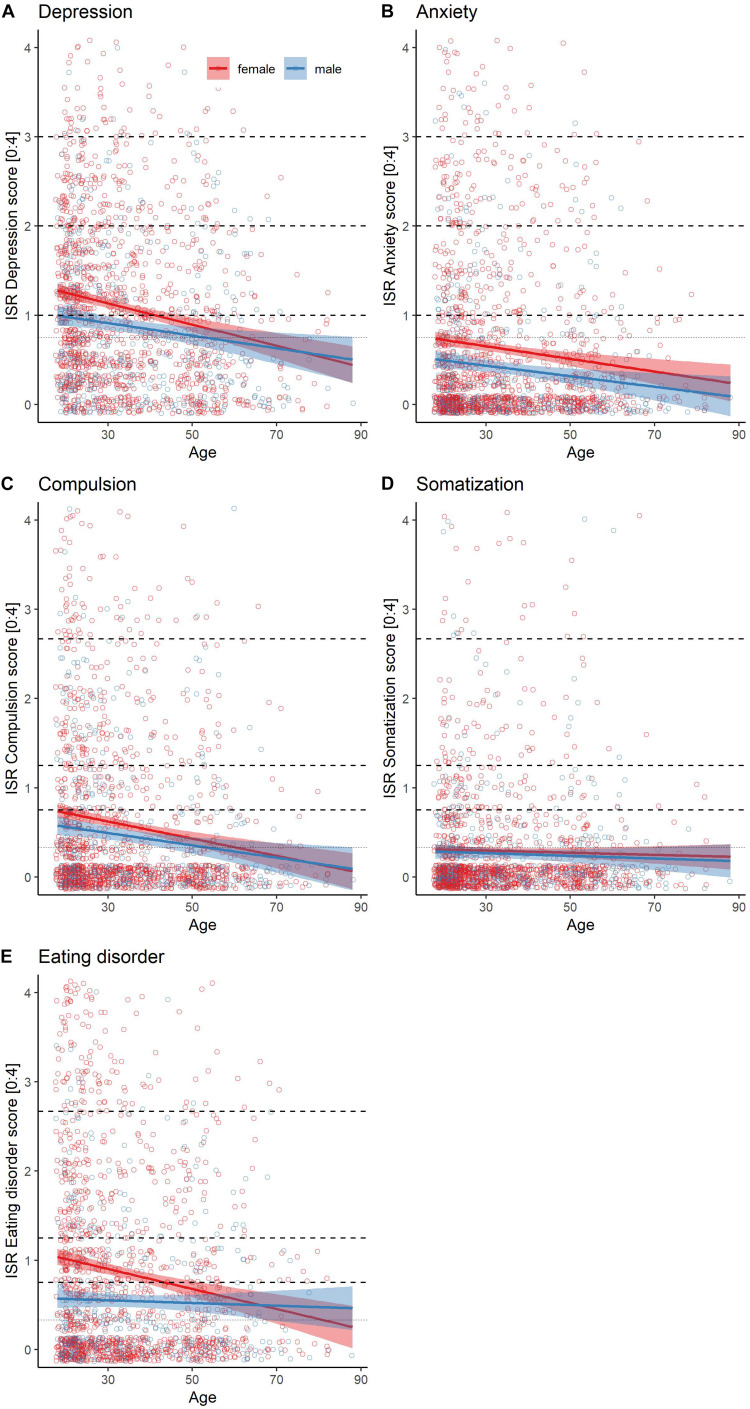
Subscale scores as a function of age and gender. Solid lines represent linear model and shaded areas confidence intervals for the subscales **(A)** Depression, **(B)** Anxiety, **(C)** Compulsion, **(D)** Somatization, and **(E)** Eating Disorder. The dashed horizontal lines represent cut-offs of symptom severity, increasing from bottom to top in mild, moderate and severe symptom levels.

**FIGURE 5:**
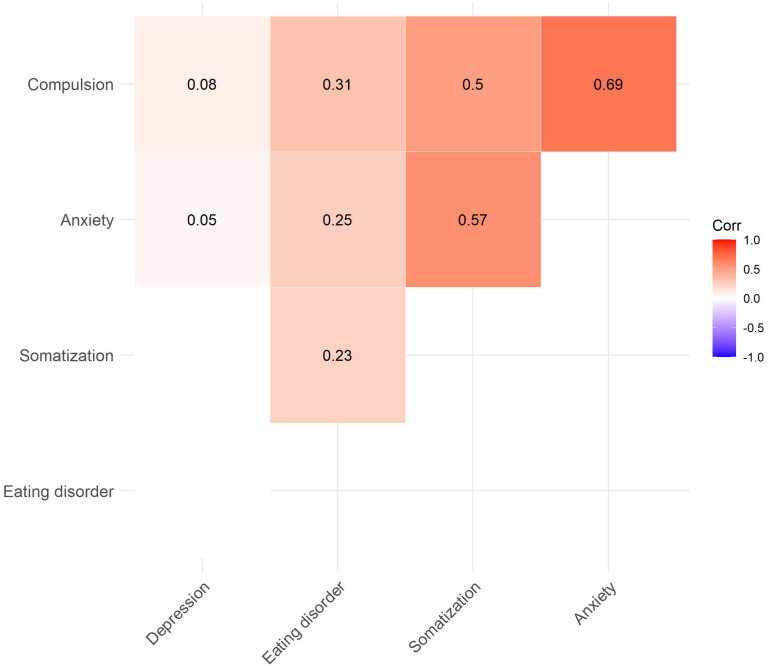
Correlation plot for the ISR subscales (only significant correlation coefficients are displayed).

We calculated linear regression models for each of the ISR symptom scales (*N* = 1,459; see Table 3 and Figure 4). For each subscale, we compared models that only considered main effects to models that included an interaction term. Except for the eating disorder subscale, we found that the added interaction term was neither a significant predictor nor did it improve the model fits. In all subscales except somatization, age was a significant predictor. For instance, for every additional year in participants’ age, the depression score decreased by 0.012 points. Similarly, male participants reported less severe symptoms than female participants (again, except for somatization). We then correlated the subscales against each other (Figure 5). Except for the eating disorder subscale, all subscales were significantly correlated to at least one other subscale, with the compulsion and anxiety subscale showing the strongest correlation.

**TABLE 3:** Results of linear regression models with age and gender predicting ISR sub-scales.

ISR Scale	Predictor	Estimate	SEM	t	p	R^2^
Depression	Age	–0.012	0.002	–6.672	< 0.001	0.044
	Gender *	–0.283	0.121	–2.339	0.019	
Anxiety	Age	–0.006	0.001	–4.444	< 0.001	0.0254
	Gender *	–0.183	0.045	-4.072	< 0.001	
Compulsion	Age	–0.009	0.001	–6.171	< 0.001	0.029
	Gender *	0.109	0.046	-2.353	0.018	
Somatization	Age	–0.001	0.001	–1.279	0.201	0.001
	Gender *	–0.012	0.034	–0.367	0.713	
Eating disorder **	Age	–0.011	0.002	–5.61	< 0.001	0.041
	Gender *	-0.649	0.141	–4.608	< 0.001	
	Age x Gender	0.009	0.003	2.692	<0.01	

*Gender dummy coded to female = 0 and male = 1; **Model fit (AIC) increased significantly by adding the interaction term, *F*(1, 1455) = 7.245, *p* < 0.01.

### State of Residence

There were no significant differences in average symptom severity for depression, compulsion, anxiety, somatization or eating disorder symptoms between Lower Saxony and Bavaria (all *p* > 0.155). Average male scores were at *M* = 0.79 (*SD* = 0.72) in Lower Saxony and *M* = 0.87 (*SD* = 0.80) in Bavaria, average female scores were *M* = 1.01 (*SD* = 0.90) in Lower Saxony and *M* = 0.90 (*SD* = 0.78) in Bavaria. For eating disorders, average scores were at *M* = 0.75 (*SD* = 1.00) in Lower Saxony and *M* = 0.76 (*SD* = 1.03) in Bavaria. Average male scores were at *M* = 0.46 (*SD* = 0.70) in Lower Saxony and *M* = 0.53 (*SD* = 0.86) in Bavaria, average female scores were *M* = 0.88 (*SD* = 1.08) in Lower Saxony and *M* = 0.85 (*SD* = 1.08) in Bavaria. Means and standard deviations can be found in Table 4.

**TABLE 4:** ISR subscales and total scores for all samples, separated by gender.

Variable	Survey sample (resampled)		Reference sample		Bavarian sample		Lower Saxony sample	
	*N* = 875		*N* = 2512		*N* = 276		*N* = 501	
	*M*	*SD*	*M*	*SD*	*M*	*SD*	*M*	*SD*
**Depression scores**	0.91	0.83	0.54	0.69	0.89	0.78	0.93	0.85
Male	0.81	0.78	–	–	0.87	0.80	0.79	0.72
Female	1.00	0.87	–	–	0.90	0.78	1.01	0.90
**Compulsion scores**	0.47	0.81	0.32	0.57	0.41	0.78	0.51	0.84
Male	0.39	0.72	–	–	0.39	0.76	0.42	0.71
Female	0.55	0.88	–	–	0.42	0.79	0.56	0.89
**Anxiety scores**	0.46	0.77	0.45	0.66	0.37	0.63	0.49	0.79
Male	0.34	0.63	–	–	0.30	0.53	0.35	0.57
Female	0.57	0.86	–	–	0.40	0.66	0.56	0.87
**Somatization scores**	0.27	0.64	0.35	0.60	0.24	0.57	0.26	0.61
Male	0.25	0.59	–	–	0.24	0.63	0.24	0.51
female	0.29	0.68	–	–	0.25	0.55	0.27	0.65
**Eating disorder scores**	0.71	0.95	0.52	0.76	0.76	1.03	0.75	1.00
Male	0.52	0.75	–	–	0.53	0.86	0.46	0.70
Female	0.88	1.07	–	–	0.85	1.08	0.88	1.08
**Sum scores**	0.53	0.55	0.40	0.45	0.49	0.47	0.55	0.57
Male	0.45	0.49	–	–	0.44	0.43	0.44	0.45
Female	0.60	0.59	–	–	0.51	0.48	0.60	0.60

*Means (*M*) and Standard Deviations (*SD*s) of the ISR subscale scores and total score. For the reference sample, gender-specific values were not available.*

## Discussion

The aim of this study was to contribute to a deeper understanding of the psychological burden associated with restrictions taken by German federal state governments as a consequence of the COVID-19 pandemic with a specific focus on depressive symptoms. By providing information concerning psychological well-being during a period of unprecedented conditions (COVID-19, lockdown measures), we aimed to support an informational base for the development of prevention and recovery action. In comparison with reference data (Tritt et al., 2010) from prior to the current outbreak, as expected, our results suggest a high prevalence of depressive symptoms, but also of compulsory and eating disorder symptoms. It appears that the prevalence of anxiety symptoms was the same in both samples, while the prevalence of somatization symptoms was higher in the reference sample.

### Findings on Symptom Prevalence

A general increase in the rates of depressive symptoms during a pandemic would be in line with previous studies conducted before the coronavirus outbreak (Hawryluck et al., 2004; Yip et al., 2010; Brooks et al., 2020) and with other prevalence studies conducted during the COVID-19 pandemic (Bäuerle et al., 2020b; Pieh et al., 2020; Rossi et al., 2020; Wang et al., 2021) as well as longitudinal studies (Castellini et al., 2021). With regard to our exploratory findings, starting with eating disorder symptoms, two studies investigating reactions of formerly diagnosed patients also reported worsening of eating disorder symptoms during lockdown in European samples (Fernández-Aranda et al., 2020; Robertson et al., 2021), studies targeting eating disorder symptoms in the general population during the COVID-19 crisis are still scarce to our knowledge: One Australian study showed an increase in restricting and binge eating behaviors (Phillipou et al., 2020). Furthermore, increases in unhealthy eating behavior became apparent: One study conducted in Italy found a weight gain in 48.6% of the population (Di Renzo et al., 2020) and an international online survey found decreases in physical activity and more unhealthy food consumption patterns (Ammar et al., 2020). There are not many studies with a focus on compulsive symptoms, however, in former patients (Jelinek et al., 2021) as well as adolescents and children (Tanir et al., 2020), increases in symptomatology have been documented. Interestingly, our study did not find elevated anxiety symptoms, which is contradictory to the results of, e.g., Li et al. (2020) and Zhu et al. (2020). However, other studies also showed no increase in anxiety symptoms in China (e.g., Wang et al., 2020) and in a longitudinal study in the Netherlands (Pan et al., 2021). Higher anxiety levels have been shown to be related to lower social capital (Xiao et al., 2020), to poor mental health (Wang et al., 2021) and to COVID-19-infections (Özdin and Bayrak Özdin, 2020) while living in urban areas and a steady family income served as protection from elevated anxiety levels (Cao et al., 2020). As most people in Germany have comparably high financial security, the degree of COVID-19-infections in our sample was low and only mentally healthy participants were included in this study, our sample might therefore not have been very vulnerable to experience anxiety. Most surprising was the low prevalence of somatization symptoms. When looking at the intercorrelations of the scales, it was interesting to note that this study showed low associations between the depressive subscale and the other scales, where other studies show high associations (e.g., Olfson et al., 2017). However, one study recently demonstrated that there are specific risk factors, not only predicting elevated psychopathological symptoms during the COVID-19 pandemic, but also comorbidities (Palgi et al., 2020). This study showed that loneliness predicted the comorbidity between anxiety and depressive symptoms. Furthermore, one could assume, that the increase in depressive symptoms was a normal rather than a psychopathological reaction to a loss of incentives, social support, ease of life, and very specific to the restrictions following the COVID-19 pandemic and therefore not associated with other psychopathologies.

### Findings on Symptom Predictors

Regarding gender and age, some differences in the rates and severity of psychological burden were found: Females showed higher rates of all scales except for somatization, which was not surprising considering that the general risk of those disorders is higher for this group (Sepulveda et al., 2008; Busch et al., 2013; Grobe et al., 2018). Females might also be especially vulnerable during the COVID-19 pandemic because they often tend to work in fields that were most affected by the pandemic, they are also more likely to be responsible for childcare which might increase worrying behaviors (Alon et al., 2020; Niedzwiedz et al., 2021). Furthermore, lockdown increased risks of domestic violence, which usually affects women more often (Alon et al., 2020).

Our results suggest, that younger people have higher symptoms in all symptom subscales than older participants, except for the somatization scale. This supports previous findings (Bäuerle et al., 2020b; Pieh et al., 2020) that mental health of young adults and women was significantly more burdened by the COVID-19 pandemic and the lockdown. One explanation could be that older people often have better emotion regulation skills (Charles, 2010) that help coping with the psychological effects of the pandemic (Barber and Kim, 2021). Results from this study showed that older men exhibited less worrying related to the Corona pandemic. Pieh et al. (2020) assumed that younger people are usually more likely to experience job insecurity and financial problems, the lockdown and the resulting restrictions might therefore have a greater impact on the daily lives of younger adults. It is important to note, that, although significant, these were small effects and our model could only explain 4% of variance.

Contrary to other studies (e.g., Benke et al., 2020), no difference in symptomatology was found between people living in Lower Saxony and Bavaria even though Bavaria had a stay-at-home order and Lower Saxony did not. This result, however, is consistent with data collected conducted prior to the pandemic, where no significant difference in prevalence rates for depression in adults was found between these two states regardless of gender (Bretschneider et al., 2017). Therefore, it can be concluded that a voluntary stay-at-home order in comparison with a forced stay-at-home order did not have an impact on differences in mental health symptoms in our sample.

### Limitations

There are several limitations to this study. First, any observations made here are purely correlational. There were neither baseline measures nor any follow-up measures conducted and therefore no statements about the development of symptoms or long-term effects can yet be made. Second, the data presented here was collected from a convenience sample. Therefore, even despite our resampling efforts, the sample reported on here, likely differs from the reference sample (Tritt et al., 2010). As the study was mainly conducted with residents of Lower Saxony and Bavaria, it is unclear if the data can be generalized to Germany’s population as a whole. In addition, the reference sample used to establish differences in symptom rates, is ten years old (Tritt et al., 2010). Since then, an increase in depressive disorders is likely, as incidence rates for depressive disorders in Germany have increased (see Kaufmännische Krankenkasse., 2020, May 25). Our study therefore cannot clearly state whether the high symptom prevalence is due to the restrictions or due to a general increase in symptoms during the last decade. Second, despite the described differences in lockdown measures between the two German states, in both states restrictions were imposed. Therefore, the study can mainly make the specific point that a forced stay-at-home order did not have an impact on psychological burden compared to a voluntary stay-at-home order.

### Implications

Still, the results of this study complement the results of other research during the first weeks after restrictions: It matches the results of comparable studies in other countries in terms of depressive symptoms (e.g., Pieh et al., 2020) and provides interesting new results concerning high prevalence of eating disorder and compulsive symptoms, especially for younger and female adults. As the ISR has been validated with a large sample size and shows good quality criteria, it serves as a good measure of psychological burden, so the data here can be considered as a screening. The current study could therefore be interpreted as a warning sign. The duration and causes of psychological burden should be further investigated, and countermeasures should be taken. In a nationwide lockdown, online mental health counseling is a far-reaching method to help many people (Dan, 2020). One helpful prevention measure is to educate the public on what mental health issues they may face during times of isolation. It is useful to provide tips on how people can handle individual situations and suffering, such as loneliness, in order to manage these situations and minimize suffering (Hiremath et al., 2020). Tele-mental-health services, online platforms and social media are helpful for support and care during mental health crises in the Corona pandemic, especially in rural areas (Zhou et al., 2020). For instance, the e-mental health intervention ^‵^CoPE It^‵^ is a low-threshold approach to support people with mental distress during the Corona pandemic (Bäuerle et al., 2020a). In Wuhan, a free psychological counseling service was offered online to reduce people’s distress (Dan, 2020). Employers in Canada supported their employees by dropping mental health fees, providing support and educating them about mental health (Ho et al., 2020). Furthermore, various online mental health services can be taken as preventive measures to identify at-risk groups (Liu et al., 2020). Still, for those developing mental disorders face-to-face therapies should be available. Sports (Pieh et al., 2020) as well as pursuing new projects or hobbies at home (de Quervain et al., 2020) seemed to function as resilience factors.

## Conclusion

Even though this study provides merely a snapshot, due to a relatively large sample size, this dataset can contribute to a better overview of the psychological burden on mentally healthy participants during the COVID-19 pandemic in parts of Germany. We conclude that the prevalence of several symptoms, among them eating disorder and compulsion symptoms, but especially depressive symptoms in our sample was high during the first weeks after the restrictions in Germany. While the level of restrictiveness had no impact on mental health, young age and female gender seemed to slightly increase psychological burden.

## Data Availability Statement

The datasets generated for this study are available on request to the corresponding author.

## Ethics Statement

The study was approved by the Ethics Committee of the Department of Psychology at the PFH – Private University of Applied Science, Göttingen (Ethics application number: 251982). The patients/participants provided their written informed consent to participate in this study.

## Author Contributions

IS, MK, and YS: concept, manuscript, and data. AE, SS, and RL: concept and manuscript. ML: data and manuscript. SR and TT: data. JB and MM: manuscript. All authors contributed to the article and approved the submitted version.

## Conflict of Interest

The authors declare that the research was conducted in the absence of any commercial or financial relationships that could be construed as a potential conflict of interest.

## Publisher’s Note

All claims expressed in this article are solely those of the authors and do not necessarily represent those of their affiliated organizations, or those of the publisher, the editors and the reviewers. Any product that may be evaluated in this article, or claim that may be made by its manufacturer, is not guaranteed or endorsed by the publisher.
